# Isotope-labeling in situ derivatization and HS-SPME arrow GC–MS/MS for simultaneous determination of fatty acids and fatty acid methyl esters in aqueous matrices

**DOI:** 10.1007/s00216-023-04930-1

**Published:** 2023-09-23

**Authors:** Lucie K. Tintrop, Jana R. Lieske-Overgrand, Kaliyani Wickneswaran, Rukiyye Abis, Ruth Brunstermann, Maik A. Jochmann, Torsten C. Schmidt

**Affiliations:** 1 Instrumental Analytical Chemistry, University of Duisburg-Essen, Universitätsstraße 5, 45141 Essen, Germany; 2 Centre for Water and Environmental Research, University of Duisburg-Essen, Universitätsstraße 5, 45141 Essen, Germany; 3 Urban Water and Waste Management, Faculty of Engineering, University of Duisburg-Essen, Universitätsstraße 15, 45141 Essen, Germany; 4 IWW Water Centre, Moritzstrasse 26, 45476 Mülheim an der Ruhr, Germany

**Keywords:** Fatty acids, Fatty acid methyl esters, Isotope-labeling esterification, SPME arrow, GC–MS/MS, Water

## Abstract

Fatty acids (FAs) and fatty acid methyl esters (FAMEs) co-occur in many samples, and analysis of both substance classes is frequently of high interest. To this end, this study introduces the first method for simultaneous determination of FAs and FAMEs including fully automated solvent-free solid-phase microextraction (SPME) arrow headspace extraction combined with isotope-labeling in situ FA derivatization with deuterated methanol (CD_3_OD). By using the chromatographic isotope effect (ΔR_t_ = 0.03 min) and the + 3 m*/z* mass shift, FAs can be selectively differentiated from the FAMEs during gas chromatography tandem-mass spectrometry (GC–MS/MS) operated in the multiple reaction monitoring (MRM) aquisition mode. Additionally, an approach is presented to predict the retention times of deuterated compounds. Optimization of the derivatization conditions was accomplished by design of experiments and found to be 20 min, 50 °C, 4 v/v% CD_3_OD, and pH 2.1. During method validation, FAs and FAMEs were calibrated in different concentration ranges by standard addition in five real matrices and ultrapure water leading to good linearities and method detection limits for FAs ranging from 1–30 µg L^−1^ and for FAMEs from 0.003–0.72 µg L^−1^. FAs and FAMEs were detected in real samples from surface water, wastewater treatment plant effluent, and three different bioreactor samples and could be quantified in concentrations ranging from 2–1056 µg L^−1^ for FAs and 0.01–14 µg L^−1^ for FAMEs.

**Graphical Abstract:**

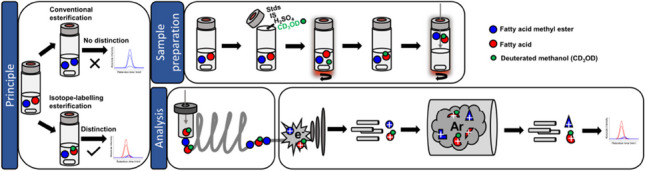

**Supplementary Information:**

The online version contains supplementary material available at 10.1007/s00216-023-04930-1.

## Introduction

### Relevance of simultaneous determination of FAs and FAMEs

Fatty acids (FAs) and fatty acid methyl esters (FAMEs) are important substance classes in science and technology including the fields of medicine [1–3], microbiology [4, 5], geochemistry [6], energy [7–9], industrial production [10], and wastewater treatment [11–13]. In general, FAs occur more frequently and in higher concentrations and are therefore the more intensively studied substance class compared with FAMEs. The simultaneous analysis of FAs and FAMEs is advantageous for various applications, as they are related, can be converted into each other, and often appear together. Some examples of application fields for the simultaneous determination of FAs and FAMEs are briefly mentioned below.

FAs are present in healthy organ tissues, however, metabolic pathways have been discovered in which tissue FAs are partly transformed into FAMEs after methanol intoxication [14]. In humans, drastically increased FAME levels can be found in tissues in case of long-term alcohol abuse [3]. A similar phenomenon can be observed in insects such as *O. furnacalis*, which can be a target of this process after plant-defense-based methanol intoxication [15]. Some defense mechanisms of microorganisms and plants are based on the production of antimicrobial FAMEs or FAs, which show an inhibitory effect [16, 17]. FAs are known to interfere with relevant biological processes, especially anaerobic digestions as in wastewater and digested sludge treatment, biodiesel, and biogas production [17–19]. For example, long-chain FAs were found to promote the growth of specific microorganisms such as *M. parvicella* which causes serious problems such as bulking and filamentous foaming in activated sludge systems [12]. In biodiesel or biogas production, lipids are often used as a source to establish the formation of desired products or as feed for microorganisms [20, 21]. In biodiesel production processes, the lipids produced in the bioreactor are converted to methyl esters after extraction by the addition of methanol for methyl esterification [22]. In another study, *E. coli* was modified to be able to directly synthesize FAMEs for biodiesel from free FAs by utilizing a bacterial fatty acid methyltransferase [7].

In all of the above-mentioned applications, it is an advantage to be able to use a simultaneous FA and FAME method to determine actual values and/or to monitor the effectiveness of the conversion processes over time. Further advantages result from the time, resources, and cost savings of the simultaneous analysis compared to two individual analyses.

### Analysis of FAs and FAMEs

Gas chromatography is a common procedure to investigate FAs, their derivatives, and FAMEs, which has been described in many studies [1, 6, 9, 12, 23]. When analyzing FAs a derivatization is recommended due to several problems that occur in the analysis of underivatized fatty acids such as low sensitivity, reproducibility and recovery [24]. In addition, long-chain FAs tend to decompose at high temperatures. In general, derivatization reactions are performed in solvent, but for aqueous samples, this would require sample preparation with an extra solvent-consuming extraction step such as solid-phase extraction (SPE) or liquid–liquid extraction (LLE) and additional steps such as solvent evaporation and reconstitution. Although SPE and LLE are still used to a large extent, their environmental impact and greenness evaluations are poor and they should be avoided as equal or even better-performing alternatives already exist. To be able to apply the derivatization to aqueous samples, it should be possible to integrate the derivatization reaction into the sample preparation process without much additional effort. This can be utilized by in situ derivatization, which has the advantage that it does not require additional time-consuming steps leading to analyte loss and it can be performed directly in the sample, being compatible with various matrices. Additionally, in situ derivatization can be combined with solvent-free and miniaturized microextraction techniques such as solid-phase microextraction, and both processes can be integrated into automated systems. Alkylation, esterification, and amide formation can be used for in situ derivatization of fatty acids, with esterification being the method with the least changes to the molecule [24]. Formation of methyl or ethyl ester derivatives of carboxylic acids in aqueous samples can be achieved with dimethyl sulfate [25, 26], diethyl sulfate [27], or alcohols in combination with a mineral acid [4]. In recent years, the use of highly toxic reagents, including dimethyl sulfate and diethyl sulfate, has become increasingly unpopular, as it is a risk to health (mutagenic and cancerogenic) and the environment. As a result, the demand for environmentally friendly, green procedures has become higher. Moreover, alcohols and mineral acids are easy to handle and available in almost every laboratory.

However, so far no method capable of analyzing FAs and FAMEs in one run has been described in literature. One reason for this may be the classically utilized derivatization technique for FAs in GC analysis: methyl esterification, which makes it impossible to distinguish the FAs and FAMEs. Here, a method is presented which allows the simultaneous detection of derivatized FAs and FAMEs. The novel approach presented in this study uses deuterated methanol (CD_3_OD) and H_2_SO_4_ to transform the FAs in situ directly in the aqueous sample resulting in a consecutive mass shift of 3 m/z and an inverse chromatographic isotope effect, which was determined by GC–MS/MS. The chromatographic isotope effect was observed and used for more reliable compound identification, therefore its characteristics are briefly mentioned in the following [28–30]. Many conventional columns are not able to separate isotopologues, mostly due to unsuitable polarity. Moreover, for nonpolar columns usually an inverse isotope effect, where the heavier isotopologue is eluted before its counterpart, and for polar columns a normal isotope effect, where the heavier isotopologue is eluted after its counterpart, is observed [30]. Meier-Augenstein suggested that the lower retention time of the heavier isotopologues can not be assigned to differences in vapor pressure because the lighter molecule would have an earlier elution due to higher vapor pressure. Since the opposite is observed, the earlier elution of the isotopologues has to be the result of weaker solute-stationary phase interactions, mainly induced by Van-der-Waals dispersion forces and caused by a lower molecular volume of the deuterated molecule. The lower molecular volume is a result of higher C-D bond strength reducing the bond length and zero-point vibrational energy [31, 32].

The derivatization conditions were optimized using a design of experiment Box-Behnken model. With the application of headspace solid-phase microextraction (SPME) arrow in this study, the extraction step could be simplified, shortened, and is solvent-free. SPME arrow, a further developed SPME, enhances mechanical stability and phase volume resulting in higher sensitivity. Compared to solvent-consuming extraction methods, such as LLE and SPE, SPME is easier to automate, applicable to aqueous samples, and follows terms of green sample preparation [33, 34].

## Material and methods

### Reagents and materials

Hexanoic acid (C6:0, ≥ 98%), octanoic acid (C8:0, ≥ 98%), nonanoic acid (C9:0, ≥ 99.5%), decanoic acid (C10:0, ≥ 99.5%), undecanoic acid (C11:0, ≥ 98%), dodecanoic acid (C12:0, ≥ 99.5%), tridecanoic acid (C13:0, ≥ 98%), tetradecanoic acid (C14:0, ≥ 99.5%), pentadecanoic acid (C15:0, ≥ 99%), hexadecenoic acid (C16:0, ≥ 99%), isotope-labeled hexadecenoic acid d_31_ (d_31_-C16:0, ≥ 99%), heptadecanoic acid (C17:0, ≥ 98%), octadecanoic acid (C18:0, ≥ 98.5%), nonadecanoic acid (C19:0, ≥ 98%), eicosanoic acid (C20:0, ≥ 99%), heneicosanoic acid (C21:0, ≥ 99%), doeicosanoic acid (C22:0, ≥ 99%), *cis*-9-hexadecenoic acid (C16:1-c9, ≥ 98.5%), *cis*-9-octadecenoic acid (C18:1-c9, ≥ 99%), *trans*-9-octadecenoic acid (C18:1-t9, ≥ 99.0%), *cis*-9, 12-octadecadienoic acid (C18:2-c9-12, ≥ 99%), *cis*-9,12,15-octadecatrienoic acid (C18:3-c9-12–15, ≥ 99%), *cis*-6,12,15-octadecatrienoic acid (C18:3-c6-9–12, ≥ 99%), *cis*-5,8,11,15,17-eicosapentanoic acid (C20:5-c5-8–11-15–17, ≥ 98.5%), heptanoic acid methyl ester (C7:0Me), nonanoic acid methyl ester (C9:0Me, ≥ 99.8%), isotope-labeled heptadecenoic acid methyl ester d_33_ (d_33_-C17:0Me, ≥ 97.5%) and 37-component FAME mix in dichloromethane with varying concentrations of 200–600 mg L^−1^ were purchased from Sigma Aldrich (Steinheim, Germany), and heptanoic acid (C7:0, ≥ 98%) from Fisher Scientific (Loughborough, United Kingdom). The derivatization reagents istotope-labeled methanol (CD_3_OD, ≥ 99.8%) and sulfuric acid (H_2_SO_4_, ≥ 95%) were purchased from Deutero (Kastellaun, Germany) and Fisher Scientific (Loughborough, United Kingdom), respectively. Stock solutions (1 g L^−1^) were prepared in chloroform (≥ 99.8%, Sigma Aldrich, Steinheim, Germany) and stored in the fridge at 4 °C. Ultrapure water was purchased from Bi-Distillation apparatus (Bi 18E with quarz glass, Quarzglas QCS, Germany). With the FA stock solutions a FA mix was prepared in chloroform with a concentration of 40 mg L^−1^ for each FA. The 37-component FAME mix with varying concentrations of 200–600 mg L^−1^ for each FAME was diluted 1:100 in chloroform to 2–6 mg L^−1^. Another FAME mix was prepared in a concentration of 2 mg L^−1^, withC7:0Me and C9:0Me, because they were missing in the 37-component FAME mix. FAs are transformed to isotope-labeled FAMEs during derivatization and are referred to as d_3_-FAMEs throughout this work, therefore the suffix “d_3_” is added to the abbreviations, e.g. C6:0Me-d_3_.

### GC MS/MS analysis

The simultaneous analysis of FAMEs and FAs was done by using a GC 2010 with a MS/MS TQ8040 (Shimadzu Deutschland GmbH, Duisburg, Germany) operating in MRM mode. The MRM settings are shown in Table S1 in ESM. Separation of the analytes was performed by a Zebron ZB-FAME capillary column (30 m × 0.25 mm × 0.20 µm, Phenomenex, Torrance, USA) for which the oven temperature program started at 40 °C, held for 5 min, and was raised with a rate of 5 °C min^−1^ to 210 °C, held for 5 min. Helium (5.0, AirLiquide, Oberhausen, Germany) was used as carrier gas with a column flow of 1.8 mL min^−1^, and argon (5.0, AirLiquide, Oberhausen, Germany) as collision gas. Injection of the analytes was done by splitless thermal desorption from the SPME arrow fiber by 250 °C for 4 min using a SPME arrow liner (1.8 mm × 5 mm × 95 mm, Topaz liner, Restek, Bad Homburg, Germany) and a wider injector needle guide for SPME arrows (Shimadzu Deutschland GmbH, Duisburg, Germany). The transfer line and ion source temperature were set to 180 °C and a solvent cut of 5 min was applied. Electron ionization (EI) was conducted at standard 70 eV. The analytical signal was integrated and the peak area was used as response in data evaluation. One quantifier ion was used for each analyte, which is stated in Table S1 in ESM.

### Automation of sample preparation

The sample preparation was fully automated with a robotic tool change prep and load (RTC PAL) autosampler (CTC Analytics, Zwingen, Switzerland) including the following steps: addition of standards, internal standards, and derivatization reagents; sample derivatization; analyte extraction by SPME arrow, sample injection, and SPME arrow fiber cleaning. All modules, units, and tools are shown in Table S2 in SI. Due to the robotic tool change option, the autosampler was able to change the tools during the procedure. The whole automation procedure is shown in the ESM in Table S3. The overall method time was 123 min, but the sample preparation is overlapping with the GC runtime, therefore 39 min per run can be saved resulting in a reduced run time of 84 min per sample and a sample throughput of 17 samples per day.

### Extraction procedure

The SPME arrow headspace extraction procedure for FAMEs was optimized in a previous work [35] to be an extraction time of 20 min, divinylbenzene polydimethylsiloxane (DVB-PDMS) extraction material, temperature of 70 °C, pH 2 (here: 2.1), stirring with glass stir bars at 1500 rpm and fiber cleaning by chemical cleaning in the headspace of 1 mL of Methanol (here: CD_3_OD) at 70 °C followed by thermal cleaning at 280 °C. The SPME arrow extraction was performed in the headspace of a 20-mL vial with 10 mL of sample.

### Optimization of derivatization parameters

Selection of the optimal derivatization temperature (40–90 °C), time (1–60 min), pH (2–4), and CD_3_OD content (1–5 v/v%) was done by using the Design of Experiments (DOE) application of OriginPro (Version 2022, OriginLab Corporation). A Box-Behnken model was used for optimization with four continuous factors, one center point per block, and 25 runs. The final parameters were selected by averaging the optimal parameters of all FAs. The DOE was conducted in ultrapure water spiked with a FA concentration of 40 µg L^−1^.

### Method validation

Calibration of FAs and FAMEs was performed directly in the samples’ matrix by spiking the required amount of FA mix and FAME mixes to the matrix solution. In ultrapure water, FAs were calibrated in the range of 4–120 µg L^−1^ and FAMEs were calibrated in the range of 0.2–2.8 µg L^−1^ with five concentration levels and seven replicates per concentration level. In the real samples (SW, WWTP, BR1-BR3), the quantification was performed by standard addition with four concentration levels plus the non-spiked sample (FAs: 0–80 µg L^−1^; FAMEs: 0–2.2 µg L^−1^) in triplicates per concentration level. The internal standards d_31_-C16:0Me-d_3_ and d_33_-C17:0Me were spiked to the solution in a concentration of 40 µg L^−1^ prior to extraction and derivatization. Peak area normalization using internal standards was not performed, as it was worsening the linearity, especially in the real sample. However, the internal standards were used to monitor fluctuations during the measurements and for retention time prediction. Assessment of the method detection limit (MDL) was done according to the MDL procedure for a spiked blank sample released by the US Environmental Protection Agency (Revision 2, 2016) [36]. Recovery measurements were conducted in triplicates by spiking the real samples with 80 µg L^−1^ with the FA mix and with 1.8–2.2 µg L^−1^ of the FAME mixes and matching the found concentrations to the calibration curve. The RSDs were calculated for the spiked real samples of the recovery measurements.

### Real samples

All of the real matrices were filtered with a polypropylene syringe filter with 0.45 µm pore size (BGB Analytik, Boeckten, Switzerland) to avoid the growth of microorganisms during the measurement and storage time. The abbreviations and measured dilutions are shown in Table 1. All real matrices were stored in the freezer (-18 °C). The conditions for BR1-BR3 indicate different settings of thermal aqueous digestion experiments of biowaste. The bioreactors were large-scale aqueous systems (> 500 L) operated at room temperature tested for the reusability of biowaste in terms of fatty acid production by microorganisms. Different biowaste pre-treatment conditions were applied to test the influence on the FA production. After digestion, the remaining aqueous phase was separated from solid biowaste parts by pressurized filtration. Analysis of the real samples was similar to the analysis of the calibration standards. SW and WWTP samples were measured non-diluted whereas BR1-BR3 were measured in 1:10 dilution.

**Table 1:** General information about the real samples including the used abbreviations (Abbr.) and measured dilutions

Source	Abbr.	Dilution
Surface water, lake	SW	None
Surface water, wastewater treatment plant effluent	WWTP	None
Bioreactor sample, (22 °C, 24 h)	BR 1	1:2
Bioreactor, (70 °C, 60 min)	BR 2	1:2
Bioreactor, (132 °C, 20 min)	BR 3	1:2

## Results and discussion

### GC–MS/MS analysis

#### Chromatographic isotope effect

**Fig. 1:**
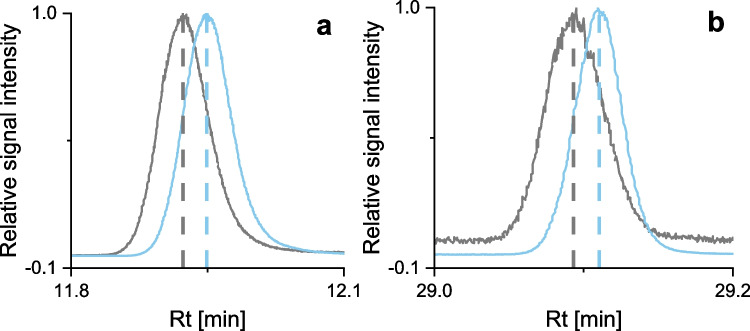
MRM chromatograms with relative signal intensity displaying the inverse chromatographic isotope effect of FAMEs (grey) and d_3_-FAMEs (blue). Exemplarily shown for **a**: C8:0Me and C8:0Me-d_3_, **b**: C20:0Me and C20:0Me-d_3_; Quantifier transitions were chosen for the visualization. Sample: UW spiked with 40 µg L^−1^ FAs and 1.1–1.6 µg L^−1^ FAMEs

When using MS/MS as in this work, there is no need for complete separation, since specific transitions can be selected in the MRM scan and full separation would require a longer GC run time. The average resolution of FAMEs and d_3_-FAMEs was found to be Rs = 0.14, which indicates that the peaks could not be fully separated by gas chromatography. Even lower temperature gradients could not achieve full separation. This may be due to the close structural similarity of FAMEs and d_3_-FAMEs and their small mass difference of 3 m/z. Figure 1 exemplarily shows the inverse chromatographic isotope effect, which is reflected by the shorter retention time of ΔR_t_ = 0.03 min (1.8 s) for d_3_-FAMEs compared to FAMEs. The chromatographic isotope effect facilitates distinguishing between FAMEs and d_3_-FAMEs in combination with different transitions in the MRM scan.

#### Retention time prediction of deuterated molecules

When a linear temperature gradient is used, the retention time of deuterated molecules can be predicted based on the number of deuterium atoms in the molecule. This approach is helpful not only for deuterated FAMEs but generally for deuterated internal standard identification because they often have no registered mass spectra. It takes time to find them in the chromatogram, especially when the molecular ion is not the base peak. In contrast, with the described retention time prediction approach, the identification of deuterated internal standards can be simplified. First of all, a fitting chromatographic linear temperature gradient has to be chosen for the separation of all desired FAMEs. After that, the retention time shift for the gradient has to be observed by measuring one FAME and d_3_-FAME solely. Thus, the retention time shift per deuterium atom ($R{t}_{shift}$) can then be calculated by subtracting the retention time of the d_3_-FAME ($R{t}_{{d}_{3}-FAME}$) from the retention time of the FAME ($R{t}_{FAME}$) and dividing this by the number of deuterium atoms of the d_3_-FAME (${n}_{d}$):

$$R{t}_{shift}= \frac{R{t}_{FAME}-R{t}_{{d}_{3}-FAME}}{{n}_{d}}$$

In this study, the retention time shift per deuterium atom resulted in average 0.009 min (0.55 s). The retention times of d_3_-FAMEs could be predicted with average 99.98% conformity (see ESM Table S4). A constant retention time shift was not expected in this manner because the relative mass difference of the molecules differs significantly with the length of the alkane chain.

#### Development of the MRM method

As the FAMEs and d_3_-FAMEs fragment strongly in the EI source and the molecular ions show a low abundance, specific EI fragments were chosen as precursors. There were two exceptions for this: for the internal standards d_31_-C16:0Me-d_3_ and d_33_-C17:0Me a higher abundance of their molecular ions was observed, consequently, their molecular ions were selected as precursors, among others. d_3_-FAME fragmentation was theoretically considered before MRM method development by calculating the d_3_-FAME fragment equivalents based on the known fragments for the FAMEs. To be able to distinguish between d_3_-FAME and FAMEs, only d_3_-FAME fragments incorporating the ester group with the observable mass shift of + 3 m/z can be taken into account. Equivalent fragment formation of + 3 m/z could be confirmed for many transitions and was helpful for the development of the MRM method. The precursors of the FAMEs were chosen based on the known fragmentations shown in Table S5 in ESM, according to Haertig et al. [37]. In all cases, the chosen d_3_-FAME and FAME precursors have two or more of the following structures: McLafferty rearrangement ion, shorter-chain methyl ester ion, and molecular ion. The McLafferty rearrangement ion was found to be 74 m/z for the FAMEs, 77 m/z for d_3_-FAMEs and the internal standard d_33_-C17:0Me, and 80 m/z for the internal standard d_31_-C16:0Me-d_3_. Collision-induced dissociation (CID) product ions of the precursors were observed and selected with help of a product ion scan. The structure of product ions could be assigned to alkyl ions, alkenyl ions, and methyl ester ions or fragment losses subtracted from the precursor ion such as methanol-, methoxy-, methoxycarbonyl losses and the losses of the before mentioned carbon alkyl- and alkenyl fragments. A detailed list of the known fragments of FAMEs and the observed fragments of d_3_-FAMEs is shown in the ESM in Table S5. In the MRM method, six specific transitions can be set per time frame, consequently, this results in three transitions, one for quantification and two for identification, for each compound. Optimal collision energies were determined by testing the CEs 5, 10, 15, 20, 25, and 30 eV and choosing the CE generating the highest peak area of the analyte. The MRM parameters are shown in Table S1 in ESM.

#### Optimization and evaluation of the derivatization procedure

The best DOE model for fitting the sum of all optimized FA responses was the full quadratic model with a correlation coefficient of 0.75, equations of all models are shown in ESM under “Equations of DOE models”. For the single FA responses, the R^2^ value of the quadratic fit ranged from 0.54–0.86. No effect of chain length nor double bonds was observed for the goodness of fit, rather the values scattered slightly over the whole range. One of the most powerful benefits of process optimization by DOE is that term combinations and significant terms can be observed, significant terms will give a p-value < 0.05. The p-values are shown in the ESM in Table S6, whereas significant values are marked green. No term has been significant for all FAs, but the pH was the most influential parameter on the FA derivatization reaction, followed by time and temperature.

**Fig. 2:**
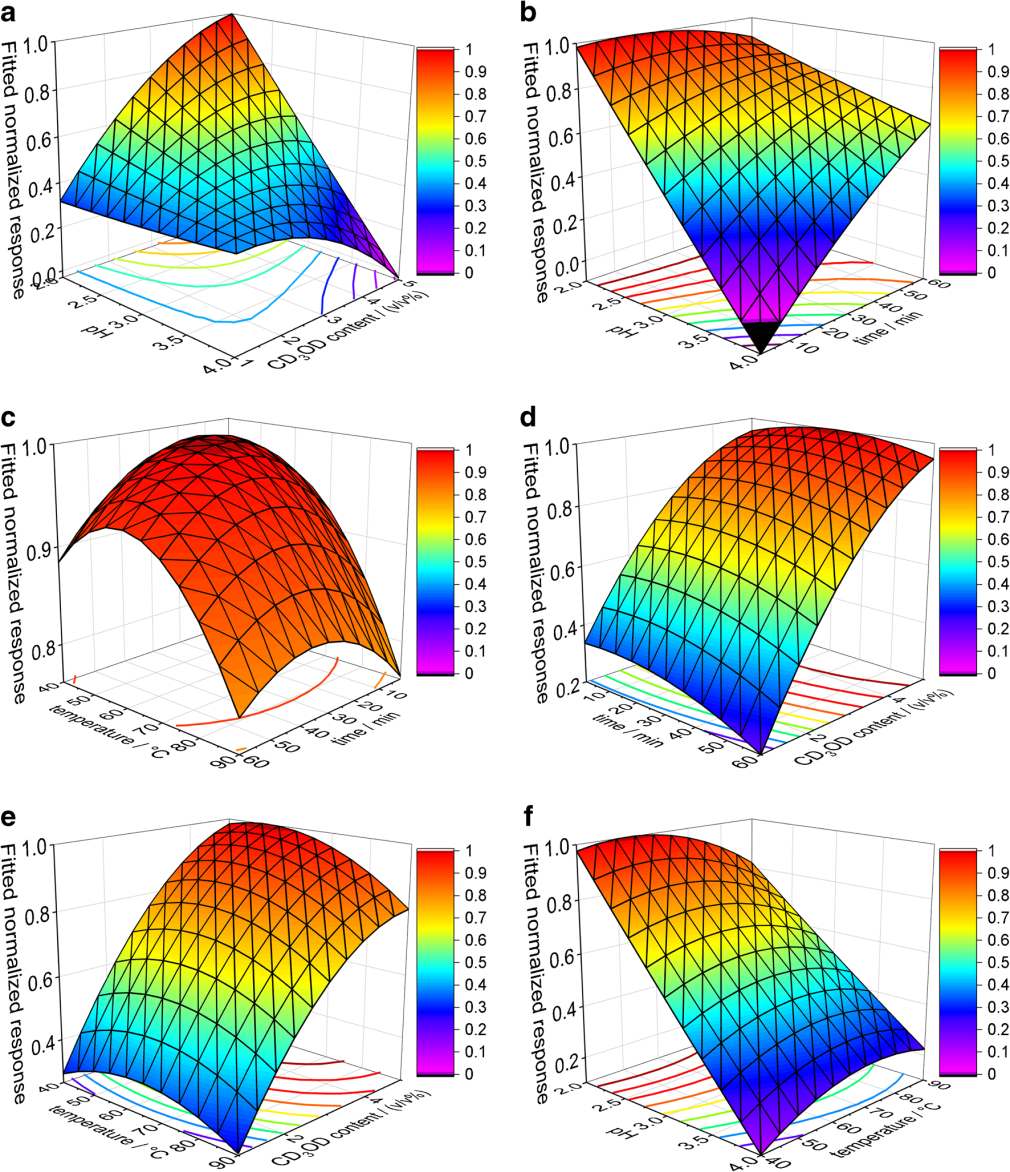
Response surface plots of the significant (**a**-**c**) and non-significant (**d**-**f**) factor combinations for derivatization parameter optimization by design of experiment. **a**: pH vs. CD_3_OD content; **b**: pH vs. time; **c**: Temperature vs. time; **d**: Time vs. CD_3_OD content; **e**: Temperature vs. CD_3_OD content; **f**: pH vs. temperature. For experimental conditions see the respective sections

The optimal derivatization parameters were determined by DOE for every single FA. Furthermore, these values have been averaged to get optimal values for the whole method (see Table S7 in ESM). The final optimal derivatization conditions were found to be pH 2.1, a temperature of 50 °C, 20 min, and a CD_3_OD content of 4 v/v%. In Fig. 2 the surface plots for the significant (a-c) and non-significant (d-f) factor combinations of all FAs are shown for which the response was cumulated. The optimal regions are displayed by red to orange color and a surface direction of 1. A preferred reaction temperature of 50 °C was unexpected as in most non-in situ derivatization reactions a higher temperature (of 60–100 °C) is chosen [12, 38]. Furthermore, a CD_3_OD content of 4 v/v% was unexpected, as one may assume that a higher CD_3_OD content would lead to higher esterification yields. However, the optimal factors for both, temperature and CD_3_OD, are assumed to have the same origin, when the whole system is considered. The SPME arrow extraction yield is substantially worsened by high solvent concentrations. During the chemical cleaning step, this behavior is utilized to remove the remaining compounds from the extraction material. However, two processes compete: 1. the increased conversion of FAs to d_3_-FAMEs at higher CD_3_OD concentrations and 2. the desorption of analytes from the fiber at higher CD_3_OD concentrations. A similar competition applies for the temperature where: 1. the higher temperature increases the concentration of CD_3_OD in the headspace of the vial and 2. the conversion of FAs to d_3_-FAMEs increases at higher temperatures.

#### Evaluation of the derivatization reaction

To evaluate the conversion ratio of FAs transformed into their d_3_-FAME derivatives, the peak area of the internal standard which is transformed during esterification d_31_-C16:0Me-d_3_ is divided with the internal standard which is not transformed during esterification d_33_-C17:0Me. This approach is intended to give an overview of the general conversion yield in an aqueous solution. In ultrapure water, an esterification conversion of only 3% was observed for the internal standards. As often discussed in literature, the esterification conversion in aqueous solution is very low as water is inhibiting the esterification [38]. The first impression is that this low conversion yield seems to be a disadvantage, but in the simultaneous method, it is an advantage since this difference allows calibration of FAs and FAMEs at different concentration ranges. Different concentration ranges are required since in most samples of natural or biological systems FAs are present in a large excess in contrast to FAMEs, which could also be confirmed by measuring different real samples. Exceptions to this are, for example, biodiesel samples, which contain predominantly FAMEs [8].

Another process that needs to be observed during the derivatization reaction is transesterification. Transesterification means that an ester group is replaced in presence of another alcohol. This process could happen for the FAMEs in the presence of CD_3_OD and could interfere with the in situ derivatization as it would produce d_3_-FAMEs. To test the derivatization for transesterification, the whole sample preparation was processed normally, with the difference that only the FAMEs were added to the solution. Transesterification seemed a likely, natural process, which cannot be avoided during this approach. However, no signal of d_3_-FAMEs was observed, therefore it can be assumed that the quantity is below the MDL. Additionally, another test was done by processing the whole sample preparation normally but adding only FAs to the solution, which showed no difference in the signals compared to a solution where FAs and FAMEs were added. Hydrogen–deuterium exchange again is a natural process that occurs to some extent, however, no reduction in the signal of FAMEs was observed when adding FAMEs and CD_3_OD, so the extent of exchange must be very low. This shows, that the method is reliably working in the targeted concentration ranges without an observed transesterification or other interferences between FAs and FAMEs.

The success of esterification strongly depends on the amounts in which the derivatization reagents are added. To estimate the required amounts, the molar excess of the derivatization reagents was calculated in Table S8 in ESM for all concentrations used in the calibration. Even at the highest concentration of 200 µg L^−1^ and the optimal pH of 2.1, an 800-fold molar excess of the H^+^ concentration over the total FA concentration is obtained. For CD_3_OD at this concentration, a 50,000-fold molar excess is present.

### Validation of the analytical method

**Fig. 3:**
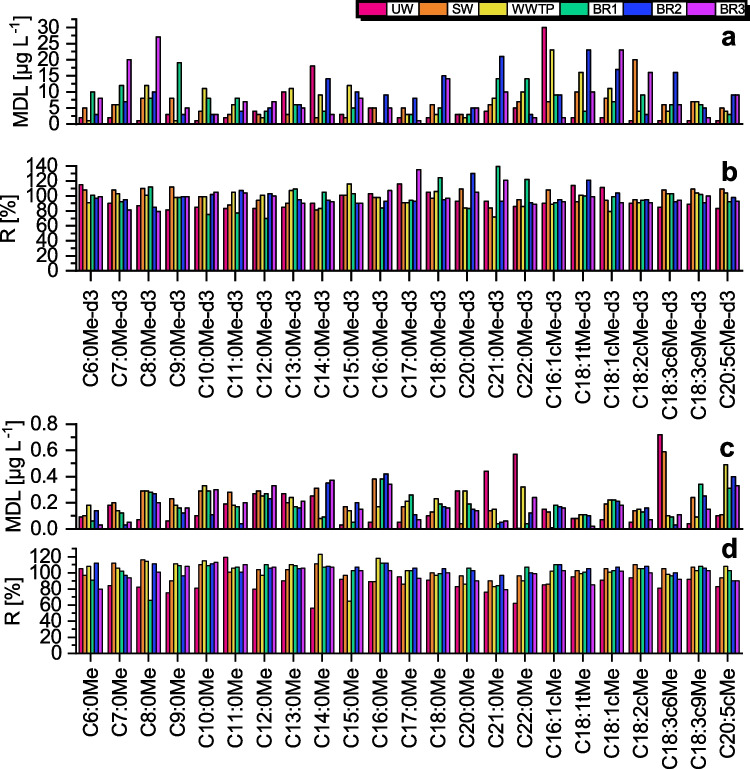
Results of method validation in different matrices with the method detection limit (MDL) and recovery (R) visualized as bar plots for d_3_-FAMEs (**a**-**b**) and FAMEs (**c**-**d**). Original values are shown in ESM in Table S10

The method detection limit (MDL) and recovery (R) are shown in Fig. 3, the linear regression functions of the calibration are shown in Table S9 in ESM and the original values are shown in Table S10 in ESM, respectively. The averaged R^2^ values for calibration in the different samples ranged from 0.9442–0.9709, which is satisfactory for a multi-component analysis in very complex matrices. It was found that the matrices of the real samples were not interfering with the esterification reaction or SPME arrow extraction as the FAs and FAMEs could be calibrated in all matrices. In addition, no strong deviation of the slopes nor a trend that could be assigned to a specific matrix was observed. The averaged MDLs for the FAs ranged from 5–9 µg L^−1^ and for the FAMEs they ranged from 0.17–0.20 µg L^−1^ (Fig. 3a and c). The MDL ranges are comparable throughout the different matrices, however, it can be noticed that the saturated FAs and FAMEs > C16:0 tended to have higher MDLs and lower R^2^ values. Lower R^2^ values and poorer MDLs for longer chain FAMEs were already found in our previous study [35]. One reason may be that the equilibrium attainment during extraction for these analytes takes longer resulting in a lower response. The recoveries show good results, ranging from 70–135% for the FAs and 56–123% for the FAMEs (Fig. 3b and d), with averaged values ranging from 83–104%.

It can be noticed, that the results for both, the FAs and FAMEs, were good, although the FAs are additionally going through the derivatization step. This shows that the derivatization works reliably and does not cause strong fluctuations. Compared to our the previous study [35], the MDLs and calibration ranges for FAMEs in this study have been higher because one quantification ion was used whereas in the previous study the total ion current of MRM transitions was used for quantification. In addition, in this study three of the six MRM channels have been used for each FA and FAME.

### Quantification of FAs and FAMEs in real samples

**Fig. 4:**
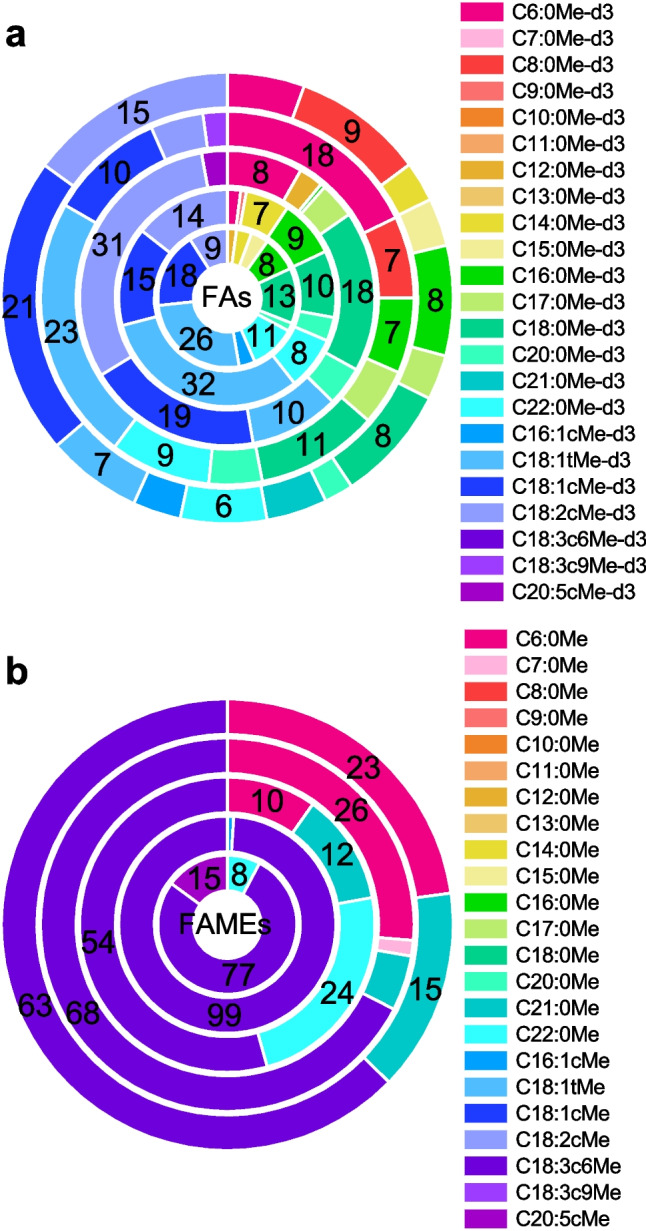
Percentual distribution of FAs (**a**) and FAMEs (**b**) in the real samples. Real samples are displayed from inside to outside: SW, WWTP, BR1, BR2, BR3. SW: surface water; WWTP: wastewater treatment plant effluent; BR1-BR3: Bioreactor samples 1–3. Only percentual values above 3% are displayed as numbers. For experimental conditions see the respective sections

Different real samples, two surface water samples, and three bioreactor samples (see Table 1) were analyzed with the developed method to test the method's applicability in real matrices and to quantify the FAs and FAMEs in these samples. Figure 4 demonstrates the percentual FA and FAME distribution in the different real samples as segmented cake plots and Table S11 in ESM displays the determined concentrations in µg L^−1^. It can be seen, that the FAs show a higher variety in the number of detected compounds than the FAMEs. The highest concentrations of FAs were found for C18:1tMe-d_3_ in SW (105 µg L^−1^), WWTP (83 µg L^−1^), BR2 (674 µg L^−1^), for C18:2cMe-d_3_ in BR1 (653 µg L^−1^) and C18:1cMe-d_3_ in BR3 (1060 µg L^−1^). Overall, the highest total FA concentration was found for BR3 (4940 µg L^−1^) demonstrating that thermal digested pre-treated biowaste at 132 °C for 20 min produces more FAs at than at 22 °C for 24 h or 70 °C for 60 min. Thus, it can be concluded that the FA concentration in the bioreactor increases with increasing pre-treatment digestion temperature, although shorter digestion times have been applied. Nevertheless, at 132 °C digestion temperature a higher variation was found, in particular, more saturated FAs were detected. The cumulated concentrations of FAs in the real samples were in the following order: WWTP < SW < BR1 < BR2 < BR3, whereas the cumulated concentrations of the surface water and wastewater treatment plant effluent (263–404 µg L^−1^) and the three bioreactor samples (2110–4940 µg L^−1^) were in the same order of magnitude, respectively.

**Fig. 5:**
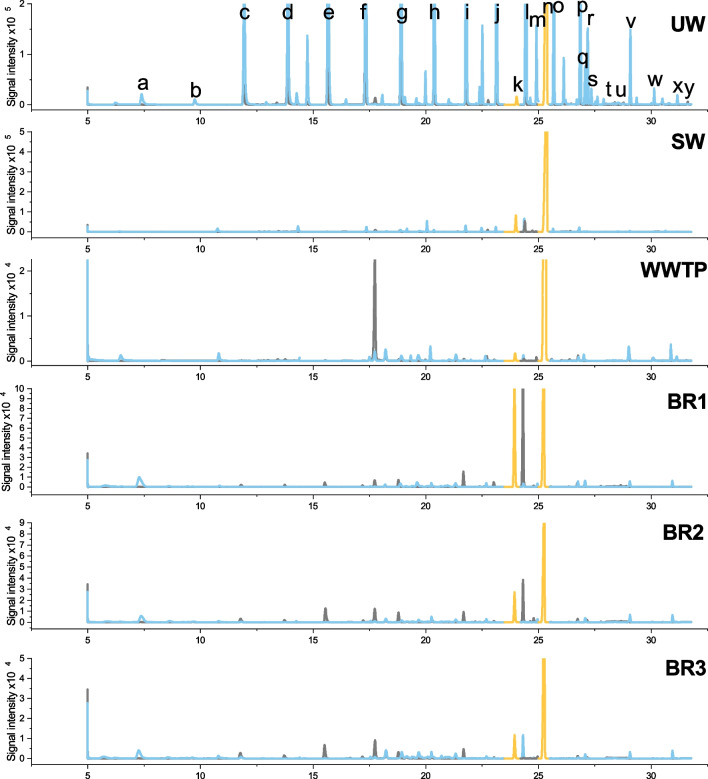
Multiple reaction monitoring (MRM) chromatograms of the d_3_-FAMEs (grey), FAMEs (blue), and internal standards (yellow) in spiked ultra pure water (UW), and non-spiked real samples from surface water (SW), wastewater treatment plant effluent (WWTP) and bioreactor 1–3 (BR1-BR3). The components are marked as follows: **a**: C6:0Me-d_3_/C6:0Me; **b**: C7:0Me-d_3_/C7:0Me; **c**: C8:0Me-d_3_/C8:0Me; **d**: C9:0Me-d_3_/C9:0Me; **e**: C10:0Me-d_3_/C10:0Me; **f**: C11:0Me-d_3_/C11:0Me; **g**: C12:0Me-d_3_/C12:0Me; **h**: C13:0Me-d_3_/C13:0Me; **i**: C14:0Me-d_3_/C14:0Me; **j**: C15:0Me-d_3_/C15:0Me; **k**: d_31_-C16:0Me-d_3_; **l**: C16:0Me-d_3_/C16:0Me; **m**: C16:1Me-d_3_/C16:1Me; **n**: d_33_-C17:0Me; **o**: C17:0Me-d_3_/C17:0Me; **p**: C18:0Me-d_3_/C18:0Me; **q**: C18:1tMe-d_3_/C18:1tMe; **r**: C18:1cMe-d_3_/C18:1cMe; **s**: C18:2cMe-d_3_/C18:2cMe; **t**: C18:3c6Me-d_3_/C18:3c6Me; **u**: C18:3c9Me-d_3_/C18:3c9Me; **v**: C20:0Me-d_3_/C20:0Me; **w**: C21:0Me-d_3_/C21:0Me; **x**: C22:0Me-d_3_/C22:0Me; **y**: C20:5cMe-d_3_/C20:5cMe

The overall variation and concentration range of FAMEs in the samples was lower than that of the FAs. The reason may be that the FAMEs are less common and lower concentrated because they are less involved in biological processes than FAs. Additionally, the bioreactor was designed to produce FAs. The highest concentrations of FAMEs were found for C18:3c6Me in all samples ranging from 0.85–14 µg L^−1^, with the highest concentration for BR2. The cumulated concentration of FAMEs in the real samples can be arranged as follows: WWTP < SW < BR1 < BR3 < BR2. Figure 5 presents exemplarily MRM chromatograms of spiked UW, SW, WWTP, and BR1-BR3.

### Analytical GREEnness Metric approach (AGREE)

**Fig. 6:**
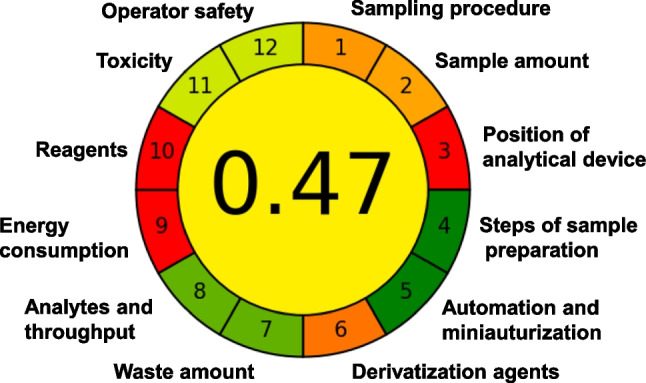
Analytical GREEnness Metric approach (AGREE) pictogram with labeled steps for the developed method

Every analytical method uses resources and energy and produces waste, but some methods have a lower environmental impact and are greener than others. For the evaluation of the method's greenness, the Analytical GREEnness Metric approach (AGREE) open-source software [39] was applied to the method's parameters. With an aspired optimal value of 1.0, the AGREE score of this method is 0.47 (see Fig. 6). This result means that the method has some parameters which should be further optimized for a lower environmental impact, but also has parameters that already have a low impact. The worst scores were obtained for the analysis being off-line, the high energy consumption of GC–MS/MS, and the type and amount of used derivatization reagents. The best scores were obtained for few steps of sample preparation, automation and miniaturization degree, waste amount, and a high number of determined analytes per run. The utilization of solvents and reagents, mostly chloroform and CD_3_OD, negatively influenced the rating in several categories. In addition, the relatively large sample amount of 10 mL increases the amount of required reagents and solvents.

It has to be taken into account that the perfect green method does not exist, but it is important to increase awareness of the environmental impact and it is also helpful for other works, which can make decisions based on the greenness scores of existing methods.

## Conclusions

The introduced method allowed simultaneous qualification and quantification of FAs and FAMEs in several aqueous matrices ranging in complexity. Due to the isotope-labeling derivatization of the FAs and the resulting mass and retention time shift the method run time is fast compared to two single analyses. The in situ derivatization and SPME arrow headspace extraction allowed full automation of the whole sample preparation with an overlapping scheduled run time (including analysis) of 84 min. Consequently, 17 samples with 46 target analytes each can be analyzed in one day. The method is suitable for all samples in which FAMEs are suspected in addition to FAs. The samples analyzed here have been from natural as well as industrial systems and always contained FAs and FAMEs. Certainly, the concentration of FAMEs was mostly lower but occurred in concentration ranges that cannot a priori be ignored and could lead to false-positive determination during classical esterification with MeOH. In situ derivatization was used here as an example of an advanced automated derivatization mode, however, isotope-labeling derivatization can be used as a non-in situ derivatization as well and can easily be integrated into already existing derivatization methods, since only MeOH has to be replaced by CD_3_OD. Due to the findings of this study, it is recommended to replace MeOH with CD_3_OD in future esterifications as it gains more reliable results when FAMEs are present next to FAs. In the real samples FAs have been found in much higher concentrations (2–1056 µg L^−1^) and broader variety than FAMEs (0.01–14 µg L^−1^). The bioreactor samples contained the highest concentrations of both. The bioreactors feeded with biowaste pre-treated at 132 °C contained the highest cumulated FA concentration.

### Supplementary Information

Below is the link to the electronic supplementary material.

Supplementary file1 (PDF 477 KB)

## References

[1] Cha D, Liu M, Zeng Z, Hu X, Guan W (2009). Analysis of fatty acids in sputum from patients with pulmonary tuberculosis using gas chromatography–mass spectrometry preceded by solid-phase microextraction and post-derivatization on the fiber. J Chromatogr A..

[2] Lotti C, Rubert J, Fava F, Tuohy K, Mattivi F, Vrhovsek U (2017). Development of a fast and cost-effective gas chromatography–mass spectrometry method for the quantification of short-chain and medium-chain fatty acids in human biofluids. Anal Bioanal Chem..

[3] Emrich J, Sprung R, Sammler J, Remberg G (1997). Identification of fatty acid methyl esters (FAMEs) in postmortem tissue. A new marker of alcohol abuse?. Forensic Sci Int.

[4] Chowdhury TR, Dick RP (2012). Standardizing methylation method during phospholipid fatty acid analysis to profile soil microbial communities. J Microbiol Methods..

[5] Dong T, Yu L, Gao D, Yu X, Miao C, Zheng Y (2015). Direct quantification of fatty acids in wet microalgal and yeast biomass via a rapid in situ fatty acid methyl ester derivatization approach. Appl Microbiol Biotechnol..

[6] Kindler R, Miltner A, Thullner M, Richnow H-H, Kästner M (2009). Fate of bacterial biomass derived fatty acids in soil and their contribution to soil organic matter. Org Geochem..

[7] Nawabi P, Bauer S, Kyrpides N, Lykidis A (2011). Engineering Escherichia coli for biodiesel production utilizing a bacterial fatty acid methyltransferase. Appl Environ Microbiol..

[8] Musharraf SG, Ahmed MA, Zehra N, Kabir N, Choudhary MI, Rahman AU (2012). Biodiesel production from microalgal isolates of southern Pakistan and quantification of FAMEs by GC-MS/MS analysis. Chem Cent J.

[9] Yaşar D, Şevket G (2006). α-tocopherol and Fatty acids of Spirulina platensis biomass in Glass panel bioreactor. Pak J Biol Sci..

[10] La Nasa J, Modugno F, Aloisi M, Lluveras-Tenorio A, Bonaduce I (2018). Development of a GC/MS method for the qualitative and quantitative analysis of mixtures of free fatty acids and metal soaps in paint samples. Anal Chim Acta..

[11] Mkhize NT, Msagati TA, Mamba BB, Momba M (2014). Determination of volatile fatty acids in wastewater by solvent extraction and gas chromatography. Phys Chem Earth..

[12] Dunkel T, de León Gallegos EL, Schoensee CD, Hesse T, Jochmann M, Wingender J (2016). Evaluating the influence of wastewater composition on the growth of Microthrix parvicella by GCxGC/qMS and real-time PCR. Water Res..

[13] Quezada M, Buitron G, Moreno-Andrade I, Moreno G, Lopez-Marin LM (2007). The use of fatty acid methyl esters as biomarkers to determine aerobic, facultatively aerobic and anaerobic communities in wastewater treatment systems. FEMS Microbiol Lett.

[14] Aleryani SL, Cluette-Brown JE, Khan ZA, Hasaba H, Lopez de Heredia L, Laposata M (2005). Fatty acid methyl esters are detectable in the plasma and their presence correlates with liver dysfunction. Clin Chim Acta.

[15] Guo L, Zeng X-Y, Wang D-Y, Li G-Q (2010). Methanol metabolism in the Asian corn borer, Ostrinia furnacalis (Guenée)(Lepidoptera: Pyralidae). J Insect Physiol..

[16] Desbois AP, Smith VJ (2010). Antibacterial free fatty acids: activities, mechanisms of action and biotechnological potential. Appl Microbiol Biotechnol..

[17] Suresh A, Praveenkumar R, Thangaraj R, Oscar FL, Baldev E, Dhanasekaran D (2014). Microalgal fatty acid methyl ester a new source of bioactive compounds with antimicrobial activity. Asian Pac J Trop Dis..

[18] Zonta Ž, Alves MM, Flotats X, Palatsi J (2013). Modelling inhibitory effects of long chain fatty acids in the anaerobic digestion process. Water Res..

[19] Alves MM, Pereira MA, Sousa DZ, Cavaleiro AJ, Picavet M, Smidt H (2009). Waste lipids to energy: how to optimize methane production from long-chain fatty acids (LCFA). Microb Biotechnol..

[20] Menon A, Lyng JG (2021). Circular bioeconomy solutions: driving anaerobic digestion of waste streams towards production of high value medium chain fatty acids. Rev Environ Sci Biotechnol.

[21] Hassan AH, Mietzel T, Brunstermann R, Schmuck S, Schoth J, Küppers M (2018). Fermentative hydrogen production from low-value substrates. World J Microbiol Biotechnol.

[22] Miao X, Wu Q (2006). Biodiesel production from heterotrophic microalgal oil. Bioresour Technol.

[23] Zhang H, Wang Z, Liu O (2015). Development and validation of a GC–FID method for quantitative analysis of oleic acid and related fatty acids. J Pharm Anal..

[24] Ferreira AMC, Laespada MEF, Pavón JLP, Cordero BM (2013). In situ aqueous derivatization as sample preparation technique for gas chromatographic determinations. J Chromatogr A..

[25] Sarrión M, Santos F, Galceran M (2000). In situ derivatization/solid-phase microextraction for the determination of haloacetic acids in water. Anal Chem..

[26] Araujo L, Wild J, Villa N, Camargo N, Cubillan D, Prieto A (2008). Determination of anti-inflammatory drugs in water samples, by in situ derivatization, solid phase microextraction and gas chromatography–mass spectrometry. Talanta..

[27] Li N, Deng C, Zhang X (2007). Determination of methylmalonic acid and glutaric acid in urine by aqueous-phase derivatization followed by headspace solid-phase microextraction and gas chromatography-mass spectrometry. J Sep Sci..

[28] Cartoni G, Liberti A, Pela A (1967). Gas chromatographic separation of polar isotopic molecules. Anal Chem..

[29] Shi B, Davis BH (1993). Gas chromatographic separation of pairs of isotopic molecules. J Chromatogr A..

[30] Thakur N, Aslani S, Armstrong DW. Evaluation of gas chromatography for the separation of a broad range of isotopic compounds. Anal Chim Acta. 2021;1165:338490; https://doi.org/10.1016/j.aca.2021.338490. 10.1016/j.aca.2021.338490 33975706

[31] Meier-Augenstein W (2002). Stable isotope analysis of fatty acids by gas chromatography–isotope ratio mass spectrometry. Anal Chim Acta..

[32] Scheiner S (2000). Calculation of isotope effects from first principles. Biochim Biophys Acta Bioenerg..

[33] Kremser A, Jochmann MA, Schmidt TC (2016). PAL SPME Arrow-evaluation of a novel solid-phase microextraction device for freely dissolved PAHs in water. Anal Bioanal Chem.

[34] Lorenzo-Parodi N, Kaziur W, Stojanovic N, Jochmann MA, Schmidt TC (2019). Solventless microextraction techniques for water analysis. Trac-Trend Anal Chem..

[35] Tintrop LK, Jochmann MA, Beesley T, Küppers M, Brunstermann R, Schmidt TC. Optimization and automation of rapid and selective analysis of fatty acid methyl esters from aqueous samples by headspace SPME arrow extraction followed by GC–MS/MS analysis. Anal Bioanal Chem. 2022; 10.1007/s00216-022-04204-2. 10.1007/s00216-022-04204-2 PMC9411252 35851411

[36] United States Environmental Protection Agency. Definition and Procedure for the Determination of the Method Detection Limit, Revision 2. 2016.

[37] Hartig C (2008). Rapid identification of fatty acid methyl esters using a multidimensional gas chromatography-mass spectrometry database. J Chromatogr A.

[38] Liu Y, Lotero E, Goodwin JG (2006). Effect of carbon chain length on esterification of carboxylic acids with methanol using acid catalysis. J Catal..

[39] Pena-Pereira F, Wojnowski W, Tobiszewski M (2020). AGREE—Analytical GREEnness metric approach and software. Anal Chem..

