# Supplementary material for: Isotope-labeling in situ derivatization and HS-SPME arrow GC–MS/MS for simultaneous determination of fatty acids and fatty acid methyl esters in aqueous matrices
Source: Anal Bioanal Chem. 2023 Sep 23;415(26):6525–36. doi: 10.1007/s00216-023-04930-1 (PMC10567957; doi:10.1007/s00216-023-04930-1)
Supplement: Supplementary file 1 — Supplementary file1 (PDF 477 KB) [file 216_2023_4930_MOESM1_ESM.pdf]

Electronic Supplementary Material for manuscript submitted to **Analytical and Bioanalytical Chemistry**

Isotope-labeling *in situ* derivatization and HS-SPME arrow GC-MS/MS for simultaneous determination of fatty acids and fatty acid methyl esters in aqueous matrices

Lucie K. Tintrop[a,b], Jana R. Lieske-Overgrand[a], Kaliyani Wickneswaran[a], Rukiyye Abis[a], Ruth Brunstermann[c], Maik A. Jochmann*[a,b], Torsten C. Schmidt[a,b,d]

[a]Instrumental Analytical Chemistry, University of Duisburg-Essen, Universitätsstraße 5, 45141 Essen, Germany

[b]Centre for Water and Environmental Research, University of Duisburg-Essen, Universitätsstrasse 5, 45141 Essen, Germany

[c]Urban Water and Waste Management, Faculty of Engineering, University of Duisburg-Essen, Universitatsstrase 15, 45141 Essen, Germany

[d]IWW Water Centre, Moritzstrasse 26, 45476 Mülheim an der Ruhr, Germany

*Corresponding author (maik.jochmann@uni-due.de)

Table of Contents

1 MRM method 3

2 Automation procedure... 8

3 Retention time prediction of deuterated molecules 9

4 Mass spectral fragmentation patterns 10

5 Equations of DOE models... 11

6 Optimal parameters and parameter dependencies obtained with DOE 12

7 Molar excess of derivatization reagents.. 13

8 Calibration and method validation.. 14

9 Quantification of FAs and FAMEs in real samples 18

References 20

1 MRM method

Table S1 MRM transitions from precursor to product ions with optimized collision energy and ion ratios for the determination of FAMEs and FAs. Time frame: chosen time frame for the given transitions; Abbr.: Abbreviation; *Quantifier ions; CE: Collision energy. For experimental conditions see the respective sections.

Time frame [min]	FAME/d_3-FAME	Abbr.	CAS No.	Retention time [min]	MRM Transitions Precursor ions > Product ions [*m/z*]	Optim. CE [eV]
5.00-8.25	Methyl hexanoate	C6:0Me	106-70-7	7.47	74>43*<br>87>55<br>101>59	5<br>10<br>5
	Methyl hexanoate-d_3	C6:0Me-d_3	-	7.43	77>44<br>90>55*<br>104>77	10<br>10<br>15
8.25-11.20	Methyl heptanoate	C7:0Me	106-73-0	9.82	74>43*<br>87>55<br>101>73	5<br>10<br>10
	Methyl heptanoate-d_3	C7:0Me-d_3	-	9.76	77>44*<br>90>55<br>104>77	10<br>10<br>15
11.20-12.70	Methyl octanoate	C8:0Me	111-11-5	11.94	74>43*<br>87>55<br>143>83	5<br>10<br>10
	Methyl octanoate-d_3	C8:0Me-d_3	-	11.91	77>44*<br>90>55<br>104>76	10<br>10<br>10
12.70-14.40	Methyl nonanoate	C9:0Me	1731-84-6	13.87	74>43*<br>87>55<br>143>101	5<br>10<br>5
	Methyl nonanoate-d_3	C9:0Me-d_3	-	13.84	77>44*<br>90>55<br>146>104	10<br>10<br>10
14.40-16.30	Methyl decanoate	C10:0Me	110-42-9	15.70	74>43<br>87>55*<br>143>83	5<br>10<br>10
	Methyl decanoate-d_3	C10:0Me-d_3	-	15.66	77>44<br>90>55*<br>146>83	10<br>10<br>10

16.30-18.00	Methyl undecanoate	C11:0Me	1731-86-8	17.35	74>43*<br>87>55<br>143>83	5<br>10<br>10
	Methyl undecanoate-d_3	C11:0Me-d_3	-	17.32	77>44*<br>90>55<br>146>83	10<br>10<br>10
18.00-19.30	Methyl dodecanoate	C12:0Me	111-82-0	18.91	74>43*<br>87>55<br>143>83	10<br>10<br>10
	Methyl dodecanoate-d_3	C12:0Me-d_3	-	18.88	77>44*<br>90>55<br>146>83	10<br>10<br>10
19.30-20.80	Methyl tridecanoate	C13:0Me	1731-88-0	20.39	74>43*<br>87>55<br>143>83	10<br>10<br>10
	Methyl tridecanoate-d_3	C13:0Me-d_3	-	20.36	77>44*<br>90>55<br>146>104	10<br>10<br>10
20.80-22.20	Methyl tetradecanoate	C14:0Me	124-10-7	21.79	74>43*<br>87>55<br>143>83	10<br>10<br>10
	Methyl tetradecanoate-d_3	C14:0Me-d_3	-	21.77	77>44*<br>90>55<br>146>104	10<br>10<br>10
22.20-23.50	Methyl pentadecanoate	C15:0Me	7132-64-1	23.13	74>43*<br>87>55<br>143>83	10<br>10<br>10
	Methyl pentadecanoate-d_3	C15:0Me-d_3	-	23.10	77>44*<br>90>55<br>146>83	10<br>10<br>10
23.50-24.20	d_{31}-Isotope-labelled methyl hexadecanoate-d_3	d_{31}-C16:0Me-d_3		24.01	80>46*<br>159>110<br>304>110	10<br>10<br>15
24.20-24.70	Methyl hexadecanoate	C16:0Me	112-39-0	24.42	74>43*<br>87>55<br>143>83	10<br>10<br>10
	Methyl hexadecanoate-d_3	C16:0Me-d_3	-	24.39	77>44*	10

					90>55<br>146>83	10<br>10
25.00-25.50	d_{33}-Isotope-labelled methyl heptadecanoate	d_{33}-C17:0Me	1219804-81-5	25.33	317>107*<br>159>94<br>188>107	25<br>10<br>5
25.50-26.00	Methyl heptadecanoate	C17:0Me	1731-92-6	25.65	74>43*<br>87>55<br>143>83	10<br>10<br>10
	Methyl heptadecanoate-d_3	C17:0Me-d_3	-	25.63	77>44<br>90>55*<br>146>83	10<br>10<br>10
26.00-26.95	Methyl octadecanoate	C18:0Me	112-61-8	26.82	74>43*<br>87>55<br>143>55	10<br>10<br>15
	Methyl octadecanoate-d_3	C18:0Me-d_3	-	26.80	77>44*<br>90>55<br>146>83	10<br>10<br>10
28.90-29.50	Methyl eicosanoate	C20:0Me	1120-28-1	29.05	74>43*<br>87>55<br>143>55	10<br>10<br>15
	Methyl eicosanoate-d_3	C20:0Me-d_3	-	29.03	77>44*<br>90>55<br>146>104	10<br>10<br>10
29.50-30.50	Methyl heneicosanoate	C21:0Me	6064-90-0	30.12	74>43*<br>87>55<br>143>55	10<br>10<br>15
	Methyl heneicosanoate-d_3	C21:0Me-d_3	-	30.09	77>44*<br>90>55<br>146>104	10<br>10<br>10
30.50-31.30	Methyl docosanoate	C22:0Me	929-77-1	31.14	74>43*<br>87>55<br>143>55	10<br>10<br>15
	Methyl docosanoate-d_3	C22:0Me-d_3	-	31.12	77>44*<br>90>55<br>146>104	10<br>10<br>10
24.70-25.00	Methyl cis-9-hexadecenoate	C16:1cMe	1120-25-8	24.90	74>43*<br>101>59<br>115>55	10<br>5<br>10

	Methyl cis-9-hexadecenoate-d_3	C16:1cMe-d_3	-	24.88	77>44*<br>104>77<br>118>91	10<br>10<br>15
26.95-27.30	Methyl trans-9-octadecenoate	C18:1tMe	112-62-9	27.05	74>43*<br>87>55<br>101>59	10<br>10<br>5
	Methyl trans-9-octadecenoate-d_3	C18:1tMe-d_3	-	27.02	77>44<br>90>55*<br>118>91	15<br>10<br>15
26.95-27.30	Methyl cis-9-octadecenoate	C18:1cMe	2777-58-4	27.16	74>43*<br>87>55<br>101>59	10<br>10<br>5
	Methyl cis-9-octadecenoate-d_3	C18:1cMe-d_3	-	27.13	77>44<br>90>55*<br>118>91	20<br>10<br>15
27.30-28.00	Methyl cis,cis-9,12-octadecadienoate	C18:2cMe	112-63-0	27.87	74>43<br>87>55*<br>115>55	15<br>10<br>10
	Methyl cis,cis-9,12-octadecadienoate-d_3	C18:2cMe-d_3	-	27.85	77>44<br>90>55*<br>118>91	20<br>10<br>15
28.00-28.60	Methyl cis,cis,cis-6,9,12-octadecatrienoate	C18:3c6Me	16326-32-2	28.39	74>45*<br>115>55<br>157>130	15<br>10<br>10
	Methyl cis,cis,cis-6,9,12-octadecatrienoate-d_3	C18:3c6Me-d_3	-	28.37	77>44<br>104>77*<br>133>91	20<br>10<br>15
28.60-28.90	Methyl cis,cis,cis Methyl cis,cis,cis-9,12,15-octadecatrienoate	C18:3c9Me	301-00-8	28.76	74>45<br>101>59<br>115>59*	15<br>10<br>10
	Methyl cis,cis,cis Methyl cis,cis,cis-9,12,15-octadecatrienoate-d_3	C18:3c9Me-d_3	-	28.74	77>44<br>104>77*<br>133>91	20<br>10<br>15
31.30-31.80	Methyl cis,cis,cis,cis,cis-5,8,11,14,17-eicosapentaenoate	C20:5cMe	2734-47-6	31.61	74>45*<br>115>71<br>157>130	10<br>10<br>10
	Methyl cis,cis,cis,cis,cis-5,8,11,14,17-eicosapentaenoate-d_3	C20:5cMe-d_3	-	31.59	77>44<br>104>77*	10<br>15

		159>88	10

2 Automation procedure

Table S2 List of the modules, tools, and suppliers used for the automation procedure.

Module/Unit/Tool	Supplier
Agitator	CTC Analytics
DeCapper	CTC Analytics
Fiber conditioning station	CTC Analytics
Heating and stirring plate with self-made heating block	IKA, Staufen, Germany
Wash station	CTC Analytics
Liquid tool 10 µL syringe	CTC Analytics
Liquid tool 100 µL syringe	CTC Analytics
Liquid tool 1000 µL syringe	CTC Analytics
SPME arrow tool	CTC Analytics

Table S3 Automation protocol displaying the different tasks, description of the tasks, and the involved objects of the task. Chronos (version 5.1.20, Axel Semrau, Sprockhoevel, Germany) was used as automation Software. FA-Mix: Fatty acid mix; FAME-Mix: 37-component FAME mix with varying concentration; M-FAME-Mix: FAME mix with missing components.

Number	Task	Description	Involved objects
1	ExecuteActivity	Set temperature of agitator	Agitator
2	DecapObject_PAL3	Remove cap from sample	DeCapper
3	Transfer	Transfer internal standard 10 µL to sample	Liquid tool 10 µL syringe
4	CleanSyringe	Cleaning syringe after IS transfer	Liquid tool 10 µL syringe; Wash station
5	Transfer	Transfer FA-Mix defined volume 1 to sample	Liquid tool 10 µL syringe or Liquid tool 100 µL syringe
6	CleanSyringe	Cleaning syringe after FA-Mix transfer	Liquid tool 10 µL syringe or Liquid tool 100 µL syringe; Wash station
7	Transfer	Transfer FAME-Mix defined volume 1 to sample	Liquid tool 10 µL syringe
8	Transfer	Transfer FAME-Mix defined volume 2 to sample	
9	CleanSyringe	Cleaning syringe after FAME-Mix transfer	Liquid tool 10 µL syringe; Wash station
10	Transfer	Transfer M-FAME-Mix defined volume 1 to sample	Liquid tool 10 µL syringe
11	Transfer	Transfer M-FAME-Mix defined volume 2 to sample	
12	CleanSyringe	Cleaning syringe after FAME-Mix transfer	Liquid tool 10 µL syringe, Wash station
13	Transfer	Transfer derivatization agent (CD_3OD) to sample	Liquid tool 1000 µL syringe
14	Transfer	Transfer H_2SO_4 (diluted in H2O) 28 µL to sample	Liquid tool 100 µL syringe
15	CapObject_PAL3	Put cap back on sample	DeCapper

16	Transport	Put sample in agitator for derivatization time	Agitator
17	Wait	Wait for sample derivatization	
18	Transport	Put wash vial with MeOH in heating block	Stirring and heating plate
19	Transport	Put sample in heating block	Stirring and heating plate
20	WaitOverlapped	Wait for sample equilibrium	Agitator
21	FiberExposure	Move to MeOH vial and expose fiber in HS	SPME arrow tool; Stirring and heating plate
22	Wait	Chemical Fiber Cleaning	SPME arrow tool; Stirring and heating plate
23	FiberAspiration	Draw in fiber	SPME arrow tool
24	Transport	Put wash vial back to initial position	Stirring and heating plate
25	FiberExposure	Move to Conditioning Station and expose fiber	SPME arrow tool; Fiber conditioning station
26	Wait	Thermal fiber cleaning	
27	FiberAspiration	Draw in fiber	
28	FiberExposure	Start sample extraction	SPME arrow tool, Stirring and heating plate
29	Wait	Enrichment	
30	FiberAspiration	Draw in fiber	
31	WaitForStartSignal	Check GC Readiness before enrichment end	-
32	FiberExposure	Move to injector and expose fiber	SPME arrow tool, GC injector
33	Wait	Desorption in injector	
34	FiberAspiration	Draw in fiber	
35	Transport	Put sample 1 back to initial position	-
36	MoveToHome	Stay at this position till next job	-
37	WaitOverlapped	GC run time	-

3 Retention time prediction of deuterated molecules

Table S4 Calculation of predicted retention time of d_3-FAMEs using the averaged retention time shift per deuterium atom and the retention time of the non-deuterated FAME. Conformity states the accordance of the predicted and actual retention time for the d_3-FAMEs. Rt: Retention time; D: deuterium;

d_3-FAME/FAME	Rt d_3-FAME [min]	Rt FAME [min]	Rt shift per D atom [min]	Pred. Rt d_3-FAME [min]	Conformity [%]
C6:0Me-d_3/C6:0Me	7.43	7.47	0.014	7.44	99.82
d-C7:0Me-d_3/C7:0Me	9.76	9.82	0.020	9.79	99.68

d-C8:0Me-d_3/C8:0Me	11.91	11.94	0.010	11.91	99.98
d-C9:0Me-d_3/C9:0Me	13.84	13.87	0.009	13.84	100.00
d-C10:0Me-d_3/C10:0Me	15.66	15.70	0.010	15.67	99.98
d-C11:0Me-d_3/C11:0Me	17.32	17.35	0.010	17.32	99.99
d-C12:0Me-d_3/C12:0Me	18.88	18.91	0.009	18.88	100.01
d-C13:0Me-d_3/C13:0Me	20.36	20.39	0.010	20.36	99.99
d-C14:0Me-d_3/C14:0Me	21.77	21.79	0.008	21.76	100.01
d-C15:0Me-d_3/C15:0Me	23.10	23.13	0.009	23.10	100.00
d-C16:0Me-d_3/C16:0Me	24.39	24.42	0.010	24.39	99.99
d-C16:1-c9Me-d_3/C16:1-c9Me	24.88	24.90	0.008	24.87	100.02
d-C17:0Me-d_3/C17:0Me	25.63	25.65	0.008	25.62	100.01
d-C18:0Me-d_3/C18:0Me	26.80	26.82	0.008	26.80	100.01
d-C18:1-t9Me-d_3/C18:1-t9Me	27.02	27.05	0.009	27.02	100.00
d-C18:1-c9Me-d_3/C18:1-c9Me	27.13	27.16	0.009	27.13	100.01
d-C18:2-c9-c12Me-d_3/C18:2-c9-12Me	27.85	27.87	0.008	27.84	100.01
d-C18:3-c6-9-12Me-d_3/C18:3-c6-9-12Me	28.37	28.39	0.007	28.36	100.02
d-C18:3-c9-12-15Me-d_3/C18:3-c9-12-15Me	28.74	28.76	0.007	28.73	100.03
d-C20:0Me-d_3/C20:0Me	29.03	29.05	0.007	29.03	100.02
d-C21:0Me-d_3/C21:0Me	30.09	30.12	0.009	30.09	100.00
d-C22:0Me-d_3/C22:0Me	31.12	31.14	0.007	31.11	100.02
d-C20:5-c-5-8-11-15-17Me-d_3/C20:5-c5-8-11-15-17Me	31.59	31.61	0.006	31.58	100.03
Mean	-	-	0.009	-	99.98

4 Mass spectral fragmentation patterns

Table S5 General mass spectral fragmentation patterns for identification of FAMEs adapted from Härtig et al. (1). Precursors and product ions were selected based on known fragmentations. M: Molecular ion

Fragments [m/z]	Identification	Derivate equivalent
43/57/71/85	Alkyl series	-
41/55/69/83	Alkenyl series	-
59	Methoxy carbonyl	62
74	McLafferty rearrangement ion	77
75	Dimethoxy methyl radical ion	
87	C3:0 Methyl ester	90
90	Cleavage at OH, H-rearrangement	
103	Cleavage at OH	
143	C7:0 methyl ester	146
199	Cleavage at C10 methyl branching site	-
M-15	Loss of methyl	-
M-18	Loss of water	-
M-29	Loss of ethyl	-
M-31	Loss of methoxy	M-34
M-32	Loss of methanol	M-35
M-43	Loss of propyl	-

M-46	Loss of ethyl + water	-
M-59	Loss of methoxy carbonyl	M-62
M-74	Loss of McLafferty fragement	M-77

5 Equations of DOE models

$$Y_{linear} = \beta_0 + \beta_1 p + \beta_2 t + \beta_3 T + \beta_4 d$$

$$Y_{linear+interactions} = \beta_0 + \beta_1 p + \beta_2 t + \beta_3 T + \beta_4 d + \beta_{12} pt + \beta_{13} pT + \beta_{14} pd + \beta_{23} tT + \beta_{24} Td + \beta_{34} Td$$

$$Y_{linear+squares} = \beta_0 + \beta_1 p + \beta_2 t + \beta_3 T + \beta_4 d + \beta_{11} p^2 + \beta_{22} t^2 + \beta_{33} T^2 + \beta_{44} d^{\,2}$$

$$Y_{full\ quadratic} = \beta_0 + \beta_1 p + \beta_2 t + \beta_3 T + \beta_4 d + \beta_{11} p^2 + \beta_{12} pt + \beta_{13} pT + \beta_{14} pd + \beta_{22} t^2 + \beta_{23} tT + \beta_{24} Td + \beta_{33} T^2 + \beta_{34} Td + \beta_{44} d^{\,2}$$

6 Optimal parameters and parameter dependencies obtained with DOE

Table S6 p-values of single and quadratic terms obtained by the full quadratic fit of the Box-Behnken model (DOE) of derivatization parameter optimization. Significant terms (<0.05) are displayed in green.

	p-values of single terms and quadratic terms												
FA	pH	T	t	CD_3OD	pH*pH	t*t	CD_3OD *CD_3OD	pH*T	pH*t	pH*CD_3OD	T*t	T* CD_3OD	t*CD_3OD
C6:0Me-d3	0.0191	0.8834	0.9718	0.1701	0.1593	0.8968	0.8301	0.2453	0.2100	0.0476	0.3531	1.0000	0.7681
C7:0Me-d3	0.0182	0.8120	0.2417	0.2706	0.0865	0.5528	0.9270	0.4999	0.0262	0.1178	0.8903	1.0000	0.7353
C8:0Me-d3	0.0213	0.8748	0.7028	0.1481	0.2556	0.6877	0.8926	0.1751	0.2194	0.0649	0.2924	0.8994	0.4680
C9:0Me-d3	0.0363	0.9603	0.4943	0.1788	0.3893	0.5763	0.9473	0.1510	0.3124	0.1359	0.2211	0.9863	0.4174
C10:0Me-d3	0.0517	0.8446	0.3808	0.2262	0.4846	0.5335	0.9650	0.1260	0.3692	0.1894	0.2311	0.9178	0.4212
C11:0Me-d3	0.0723	0.6114	0.2174	0.2662	0.5904	0.4729	0.9303	0.1002	0.4528	0.2573	0.3514	0.8434	0.3689
C12:0Me-d3	0.2571	0.0695	0.0085	0.4057	0.9792	0.0692	0.4502	0.2320	0.2492	0.2553	0.4602	0.5163	0.4335
C13:0Me-d3	0.4324	0.0063	0.0014	0.8689	0.5581	0.0210	0.2402	0.3659	0.1587	0.4385	0.0048	0.3731	0.9538
C14:0Me-d3	0.5601	0.0294	0.0048	0.4305	0.7721	0.0380	0.2390	0.3315	0.1762	0.4421	0.0095	0.7378	0.6312
C15:0Me-d3	0.4886	0.2651	0.0559	0.2454	0.6297	0.1138	0.3094	0.7004	0.2743	0.3491	0.1500	0.8844	0.4293
C16:0Me-d3	0.1622	0.7647	0.3637	0.4205	0.3550	0.2448	0.3519	0.9567	0.5500	0.3876	0.5490	0.9788	0.5500
C17:0Me-d3	0.0326	0.9276	0.7656	0.9478	0.1368	0.4237	0.8202	0.8631	0.1582	0.1364	0.8934	0.9855	0.6645
C18:0Me-d3	0.0212	0.9312	0.3556	0.3012	0.0945	0.8750	0.8183	0.7954	0.0705	0.0558	0.9888	0.9577	0.9380
C20:0Me-d3	0.0254	0.8924	0.3111	0.1955	0.1097	0.9945	0.8190	0.7932	0.0721	0.0476	0.9186	0.9725	0.9407
C21:0Me-d3	0.0332	0.9101	0.3393	0.2287	0.1627	0.8388	0.9109	0.7376	0.0688	0.0647	0.7525	0.9604	0.9418
C22:0Me-d3	0.0248	0.8353	0.2765	0.2357	0.1619	0.9421	0.9125	0.8218	0.0696	0.0550	0.7755	0.8992	0.9890
C16:1cMe-d3	0.4893	0.1608	0.0225	0.1951	0.9686	0.0518	0.3623	0.6275	0.1703	0.2379	0.0637	0.9716	0.3412
C18:1tMe-d3	0.0410	0.9134	0.5804	0.9547	0.1727	0.3709	0.9045	0.8086	0.0921	0.2098	0.9849	0.9776	0.6376
C18:1cMe-d3	0.0642	0.8798	0.9343	0.6670	0.2509	0.2953	0.8323	0.8551	0.1889	0.2961	0.9310	0.9374	0.4974
C18:2cMe-d3	0.2079	0.4018	0.1144	0.3807	0.5538	0.1430	0.3371	0.9000	0.7039	0.3926	0.3273	0.9075	0.4595
C18:3c6Me-d3	0.7151	0.1638	0.0768	0.6392	0.6493	0.0718	0.0554	0.9095	0.7068	0.5518	0.2897	0.8324	0.5899
C18:3c9Me-d3	0.4225	0.0151	0.0016	0.6811	0.5688	0.0147	0.1181	0.5147	0.4982	0.3107	0.0111	0.9818	0.6732
C20:5cMe-d3	0.0274	0.5512	0.9150	0.4183	0.5735	0.3005	0.9857	0.2934	0.1193	0.2628	0.0591	0.3936	0.7981
Total number significant terms	11	3	5	0	0	3	0	0	1	2	3	0	0
Percentage of significant terms [%]	48	13	22	0	0	13	0	0	4	9	13	0	0

Table S7 Optimal derivatization parameters for single FAs obtained with Box-Behnken model (DOE) and full quadratic fit. The parameters were averaged to determine the overall optimal parameters. pH values were averaged by the molar H^+ concentration.

FA	pH (H^+ conc. [M])	T [°C]	t [min]	CD_3OD [v/v%]
C6:0Me-d3	2.0 (10^{-2})	40.3	1.0	5.0
C7:0Me-d3	2.0 (10^{-2})	40.3	1.0	5.0
C8:0Me-d3	2.0 (10^{-2})	40.3	1.0	5.0
C9:0Me-d3	2.0 (10^{-2})	40.3	1.0	5.0
C10:0Me-d3	2.0 (10^{-2})	40.0	1.0	3.0
C11:0Me-d3	2.0 (10^{-2})	40.0	1.0	5.0
C12:0Me-d3	2.0 (10^{-2})	40.0	60.0	5.0
C13:0Me-d3	4.0 (10^{-4})	40.0	60.0	3.0
C14:0Me-d3	4.0 (10^{-4})	40.0	60.0	3.0
C15:0Me-d3	4.0 (10^{-4})	40.0	60.0	1.0
C16:0Me-d3	2.0 (10^{-4})	69.1	1.0	3.9
C17:0Me-d3	2.0 (10^{-2})	63.7	1.0	5.0
C18:0Me-d3	2.0 (10^{-2})	63.7	1.0	5.0
C20:0Me-d3	2.0 (10^{-2})	65.0	1.0	5.0
C21:0Me-d3	2.0 (10^{-2})	65.0	1.0	5.0
C22:0Me-d3	2.0 (10^{-2})	65.0	1.0	5.0
C16:1cMe-d3	2.0 (10^{-2})	40.0	60.0	1.0
C18:1tMe-d3	2.0 (10^{-2})	62.4	1.0	5.0
C18:1cMe-d3	2.0 (10^{-2})	62.4	1.0	5.0
C18:2cMe-d3	2.0 (10^{-2})	50.8	60.0	2.6
C18:3c6Me-d3	3.2 ($10^{-3.2}$)	40.0	60.0	2.6
C18:3c9Me-d3	3.6 ($10^{-3.6}$)	40.0	60.0	2.8
C20:5cMe-d3	2.0 (10^{-2})	90.0	1.0	5.0
Mean	2.1 ($10^{-2.1}$)	49.5	22.5	4.0
Final	2.1	50.0	20.0	4.0

7 Molar excess of derivatization reagents

Table S8 Calculation of the molar excess of the derivatization agents at different FA mix concentrations.

	4 µg L^{-1}	20 µg L^{-1}	40 µg L^{-1}	80 µg L^{-1}	120 µg L^{-1}
CD_3OD	$2 \cdot 10^6$	$5 \cdot 10^5$	$2 \cdot 10^5$	$1 \cdot 10^5$	$8 \cdot 10^4$
pH 2	$5 \cdot 10^4$	$1 \cdot 10^4$	$5 \cdot 10^3$	$2 \cdot 10^3$	$2 \cdot 10^3$
pH 3	$5 \cdot 10^3$	$1 \cdot 10^3$	$5 \cdot 10^2$	$2 \cdot 10^2$	$2 \cdot 10^2$
pH 4	$5 \cdot 10^2$	$1 \cdot 10^2$	$5 \cdot 10^1$	$2 \cdot 10^1$	$2 \cdot 10^1$
Final pH 2.1	$4 \cdot 10^4$	$8 \cdot 10^3$	$4 \cdot 10^3$	$2 \cdot 10^3$	$1 \cdot 10^3$

8 Calibration and method validation

Table S9 Linear regression functions with slope m and y-intercept b and R^2 for calibrations in ultrapure water (UW), surface water (SW), wastewater treatment plant outlet (WWTP), and bioreactor samples 1-3.

	UW		SW		WWTP		BR1		BR2		BR3	
**FA**	m	b	m	b	m	b	m	b	m	b	m	b
C6:0Me-d3	54	-22	34	40	20	104	103	1811	95	5002	147	4024
C7:0Me-d3	726	-3811	422	-1806	221	-672	139	754	214	1303	212	1880
C8:0Me-d3	1964	-2551	399	-1246	162	507	355	269	281	5846	284	13185
C9:0Me-d3	3072	-5217	591	-2990	181	387	183	3509	1043	2274	722	2334
C10:0Me-d3	2847	4301	279	1016	246	91	1106	-8190	1408	-412	1025	-99
C11:0Me-d3	4790	20132	688	-603	704	-2733	2915	-21827	3735	-11347	2614	-7550
C12:0Me-d3	3228	54574	585	5069	712	659	1636	9187	5465	5981	3649	7522
C13:0Me-d3	1406	41586	289	843	382	-1436	2616	-7934	3829	11923	3115	12857
C14:0Me-d3	1306	34769	236	3381	231	3971	2478	-7137	4551	19271	1835	25977
C15:0Me-d3	1088	28972	71	1200	702	-5930	1414	-5811	1911	4459	775	13135
C16:0Me-d3	725	99554	333	10975	1573	35582	4347	4365	2995	57178	1935	75478
C17:0Me-d3	333	40451	78	372	310	463	455	3818	392	5603	442	7035
C18:0Me-d3	817	26876	579	30125	924	24179	850	31610	828	25737	704	29142
C20:0Me-d3	118	8636	324	2563	304	2862	303	2758	285	3843	345	3548
C21:0Me-d3	40	3441	122	535	100	417	105	619	77	1194	74	1593
C22:0Me-d3	27	2217	34	1467	44	929	70	548	48	1236	41	1248
C16:1cMe-d3	97	3953	18	276	109	1666	204	1005	292	1341	110	1876
C18:1tMe-d3	77	1653	108	11393	130	10797	600	12418	122	8217	161	5437
C18:1cMe-d3	96	1876	124	8880	249	9654	334	13446	237	6988	61	6404
C18:2cMe-d3	10	314	15	542	16	600	16	1012	35	467	6	477
C18:3c6Me-d3	154	1756	253	-922	277	-709	374	-254	197	1570	183	948
C18:3c9Me-d3	114	1509	144	-225	176	-292	243	-164	140	861	116	56
C20:5cMe-d3	199	1492	349	-1277	384	-355	508	2985	274	1127	256	1295
**FAME**												
C6:0Me	74522	-13598	13668	-280	38007	-6326	40284	5280	24718	13651	45772	14380
C7:0Me	190854	-49711	43002	-6118	43930	-5313	44512	-3524	34561	996	49237	-336
C8:0Me	910121	-214355	143411	-36670	105580	-21161	96915	-15944	145673	-31502	192200	-34372
C9:0Me	1277187	-378815	58894	-1290	83852	-13438	109625	-14799	74489	6110	274586	-34635
C10:0Me	2573034	-608102	260061	-51498	295350	-80551	359980	-82488	353606	-20904	1059000	-271590
C11:0Me	2595071	-488079	224383	-18760	240033	-27255	398650	-51783	451136	11742	944228	-135170

C12:0Me	5041930	40064	854617	-142067	573596	-89854	1491383	-368915	1981750	-192491	3176328	-692281
C13:0Me	2553019	-144874	341121	-26796	344352	-57136	644638	-100542	970756	-7917	2196993	-297747
C14:0Me	4158998	-530539	484693	-97755	366521	-221537	272964	-13687	2291116	-347033	2859870	-582170
C15:0Me	2075896	-83723	144649	-2037	228906	-26970	181459	-6142	1362398	-135417	1459012	-83230
C16:0Me	2978994	778075	473901	-80049	227445	-7961	4780543	-1787611	3421533	-908045	3295656	-591603
C17:0Me	1443219	27969	180510	-8909	1099476	-200284	1348535	-114952	1011604	-15997	930768	55860
C18:0Me	733757	387908	1172843	-92084	1273026	-109655	1453330	-119332	1248796	-71904	1202588	3815
C20:0Me	116298	202982	379496	-4804	505759	-51747	436946	-13395	371786	2830	315299	28671
C21:0Me	32277	47097	85639	8097	81684	8443	82333	13409	84269	8410	66155	13277
C22:0Me	35020	27327	68833	5774	88864	-2545	54837	17267	64696	5010	62146	4793
C16:1cMe	268452	-28214	27514	-1288	14559	163	364408	-65452	254833	-32441	233617	-18084
C18:1tMe	115233	23434	269364	-7140	298589	-6072	357878	-10989	284194	144	218485	33651
C18:1cMe	222875	41309	248913	-31423	275725	-32812	330101	-42694	262765	-25612	242005	-5365
C18:2cMe	20073	-2031	18069	-1887	21153	-2102	26076	-2481	16954	-954	14984	340
C18:3c6Me	1805	693	41576	35170	40330	46559	74261	53804	40894	57883	40299	34877
C18:3c9Me	1483	-5	4559	-752	2679	-68	3997	-276	3212	-121	2971	-141
C20:5cMe	773	340	1452	237	1761	-36	1849	25	1121	74	1242	207

Table S10 Results of method validation in different matrices with the method detection limit (MDL) in µg L^{-1}, Recovery (R), and linear calibration curve correlation coefficient (R^2). FA: Fatty acid; FAME: Fatty acid methyl ester; UW: Ultra pure water; SW: Surface water; WWTP: Wastewater treatment plant effluent; BR1-3: Bioreactor water 1-3.

	UW			SW			WWTP			BR1			BR2			BR3		
**FA**	MDL	R [%]	R^2	MDL	R [%]	R^2	MDL	R [%]	R^2	MDL	R [%]	R^2	MDL	R [%]	R^2	MDL	R [%]	R^2
C6:0Me-d_3	2	115	0.9966	5	108	0.9276	1	91	0.9682	10	101	0.9849	3	97	0.9547	8	99	0.9877
C7:0Me-d_3	2	90	0.9829	6	108	0.9516	6	103	0.9856	12	92	0.9948	7	95	0.9868	20	81	0.9554
C8:0Me-d_3	1	87	0.9971	8	110	0.9248	12	101	0.9829	8	112	0.8854	10	85	0.9032	27	79	0.8719
C9:0Me-d_3	3	81	0.9961	8	112	0.8962	1	98	0.9898	19	98	0.9891	3	99	0.9910	5	99	0.9859
C10:0Me-d_3	1	85	0.9989	4	99	0.9843	11	99	0.9785	8	75	0.9886	3	102	0.9903	3	105	0.9786
C11:0Me-d_3	2	83	0.9954	3	88	0.9906	6	105	0.9730	8	77	0.9965	4	107	0.9608	7	104	0.9843
C12:0Me-d_3	4	83	0.9724	3	94	0.9969	2	101	0.9780	4	70	0.9983	5	103	0.9816	7	100	0.9874
C13:0Me-d_3	10	85	0.9599	3	90	0.9848	11	107	0.9592	6	109	0.9345	6	95	0.9956	5	90	0.9766

C14:0Me-d_3	18	90	0.9965	2	81	0.9727	9	83	0.9356	4	105	0.9682	14	94	0.9850	3	92	0.9530
C15:0Me-d_3	3	101	0.9940	2	101	0.9496	12	116	0.9576	5	103	0.9710	10	90	0.9985	8	90	0.9119
C16:0Me-d_3	5	103	0.9775	5	98	0.9227	0.4	98	0.9958	0.2	84	0.9820	9	93	0.9752	5	107	0.9961
C17:0Me-d_3	2	116	0.9469	5	91	0.9191	3	91	0.9183	3	94	0.9629	8	93	0.9544	1	135	0.9364
C18:0Me-d_3	2	105	0.9324	6	97	0.9948	3	106	0.9132	5	124	0.9026	15	95	0.9961	14	97	0.9636
C20:0Me-d_3	3	93	0.9674	3	109	0.9787	2	84	0.9745	3	83	0.9455	5	130	0.9004	5	105	0.9867
C21:0Me-d_3	4	93	0.9635	6	84	0.7699	8	72	0.9535	14	139	0.9168	21	93	0.9304	10	121	0.9707
C22:0Me-d_3	5	86	0.9367	7	95	0.9345	10	86	0.9250	14	122	0.9172	3	91	0.9060	2	89	0.9156
C16:1cMe-d_3	30	90	0.9968	7	108	0.9452	23	89	0.9089	9	91	0.9340	9	95	0.9951	2	92	0.9397
C18:1tMe-d_3	2	114	0.9234	10	92	0.9553	16	101	0.9769	4	100	0.9989	23	121	0.9807	10	99	0.9969
C18:1cMe-d_3	2	111	0.9170	8	94	0.9386	11	79	0.9559	7	99	0.9907	17	104	0.9836	23	91	0.8495
C18:2cMe-d_3	1	90	0.9527	20	95	0.9862	4	91	0.9191	9	94	0.9570	3	95	0.9986	16	91	0.9232
C18:3c6Me-d_3	1	85	0.9686	6	108	0.9982	4	103	0.9842	6	103	0.9843	16	92	0.9881	6	94	0.9837
C18:3c9Me-d_3	1	89	0.9731	7	109	0.9881	7	104	0.9857	6	102	0.9918	5	91	0.9497	2	100	0.9970
C20:5cMe-d_3	1	83	0.9847	5	109	0.9387	4	104	0.9844	3	92	0.9931	9	98	0.9803	9	93	0.9990
Mean	5	94	0.9709	6	99	0.9500	7	96	0.9610	7	99	0.9647	9	98	0.9690	9	98	0.9587
**FAME**																		
C6:0Me	0.09	105	0.9908	0.10	97	0.9987	0.18	108	0.9525	0.06	91	0.9886	0.14	112	0.9545	0.03	80	0.9928
C7:0Me	0.18	84	0.9610	0.20	112	0.9337	0.14	106	0.9741	0.12	102	0.9910	0.03	97	0.9899	0.05	94	0.9809
C8:0Me	0.07	82	0.9748	0.29	116	0.8893	0.29	114	0.9115	0.28	66	0.8879	0.27	111	0.9340	0.20	101	0.9638
C9:0Me	0.06	75	0.9683	0.23	90	0.9939	0.18	111	0.9402	0.16	109	0.9549	0.12	96	0.9733	0.16	108	0.9682
C10:0Me	0.10	81	0.9861	0.29	110	0.9466	0.33	115	0.8939	0.29	109	0.9424	0.11	111	0.9731	0.30	113	0.9136
C11:0Me	0.19	119	0.9928	0.28	101	0.9884	0.18	106	0.9625	0.17	107	0.9664	0.04	101	0.9797	0.20	110	0.9516
C12:0Me	0.27	80	0.9931	0.29	104	0.9719	0.25	97	0.9444	0.27	110	0.9288	0.23	106	0.9681	0.33	107	0.9516
C13:0Me	0.27	90	0.9964	0.20	104	0.9815	0.24	110	0.9452	0.17	109	0.9478	0.16	105	0.9774	0.21	106	0.9727
C14:0Me	0.25	56	0.9930	0.31	111	0.9357	0.08	123	0.8862	0.09	107	0.9475	0.35	108	0.9639	0.37	107	0.9608
C15:0Me	0.03	92	0.9987	0.17	97	0.9990	0.14	65	0.9216	0.05	103	0.9699	0.20	107	0.9735	0.15	103	0.9931
C16:0Me	0.05	89	0.9479	0.38	89	0.9192	0.17	118	0.7926	0.38	112	0.8770	0.42	112	0.9203	0.34	103	0.9620
C17:0Me	0.05	95	0.9453	0.17	86	0.9847	0.21	103	0.9336	0.26	103	0.9888	0.11	106	0.9798	0.07	93	0.9896
C18:0Me	0.10	91	0.9655	0.13	100	0.9861	0.23	97	0.9676	0.19	99	0.9834	0.17	105	0.9843	0.16	100	0.9859
C20:0Me	0.29	83	0.9269	0.04	96	0.9765	0.29	86	0.9585	0.19	106	0.9800	0.15	103	0.9810	0.14	90	0.9709
C21:0Me	0.44	76	0.9567	0.14	90	0.9860	0.15	83	0.9437	0.04	84	0.9562	0.05	97	0.9667	0.06	79	0.9466
C22:0Me	0.57	62	0.9525	0.003	96	0.9923	0.32	90	0.9745	0.04	107	0.9793	0.12	100	0.9617	0.24	99	0.9851
C16:1cMe	0.15	85	0.9945	0.13	86	0.9853	0.01	102	0.9617	0.18	110	0.9462	0.17	110	0.9504	0.16	103	0.9911
C18:1tMe	0.08	95	0.9708	0.08	103	0.9950	0.11	99	0.9947	0.11	101	0.9957	0.10	105	0.9869	0.02	85	0.9559

C18:1cMe	0.07	91	0.9489	0.19	105	0.9799	0.22	101	0.9782	0.22	103	0.9770	0.21	107	0.9730	0.18	102	0.9806
C18:2cMe	0.05	94	0.9720	0.14	110	0.9497	0.15	105	0.9802	0.13	105	0.9809	0.16	108	0.9704	0.07	100	0.9893
C18:3c6Me	0.72	81	0.9518	0.59	105	0.9277	0.10	98	0.9417	0.09	96	0.9585	0.03	100	0.9432	0.11	92	0.9567
C18:3c9Me	0.04	92	0.9744	0.24	107	0.9591	0.09	103	0.9932	0.34	108	0.9640	0.25	106	0.9791	0.15	103	0.9900
C20:5cMe	0.10	83	0.9312	0.11	94	0.9510	0.49	108	0.9640	0.31	103	0.9673	0.40	90	0.9821	0.33	90	0.9712
Mean	0.18	86	0.9693	0.20	100	0.9666	0.20	102	0.9442	0.18	102	0.9600	0.17	104	0.9681	0.18	99	0.9706

9 Quantification of FAs and FAMEs in real samples

Table S11 Results of analyte quantification in real samples.

	SW	WWTP	BR1	BR2	BR3
**FA**	c [µg L^{-1}]				
C6:0Me-d_3	Nd	5	175	526	274
C7:0Me-d_3	Nd	Nd	Nd	Nd	Nd
C8:0Me-d_3	Nd	Nd	Nd	208	465
C9:0Me-d_3	Nd	2	Nd	Nd	Nd
C10:0Me-d_3	Nd	Nd	Nd	Nd	Nd
C11:0Me-d_3	Nd	Nd	Nd	Nd	Nd
C12:0Me-d_3	9	Nd	56	Nd	Nd
C13:0Me-d_3	Nd	Nd	Nd	Nd	Nd
C14:0Me-d_3	14	17	Nd	Nd	142
C15:0Me-d_3	17	Nd	Nd	Nd	169
C16:0Me-d_3	33	23	10	191	390
C17:0Me-d_3	Nd	Nd	84	143	159
C18:0Me-d_3	52	26	372	311	414
C20:0Me-d_3	8	9	91	135	103
C21:0Me-d_3	Nd	Nd	Nd	Nd	215
C22:0Me-d_3	43	21	Nd	256	305
C16:1cMe-d_3	15	Nd	Nd	Nd	170
C18:1tMe-d_3	105	83	207	674	338
C18:1cMe-d_3	72	39	402	294	1056
C18:2cMe-d_3	36	38	653	134	741
C18:3c6Me-d_3	Nd	Nd	Nd	Nd	Nd
C18:3c9Me-d_3	Nd	Nd	Nd	62	Nd
C20:5cMe-d_3	Nd	Nd	59	Nd	Nd
Sum c [µg L^{-1}]	404	263	2109	2934	4941
**FAME**					
C6:0Me	Nd	Nd	1.3	5.5	3.1
C7:0Me	Nd	Nd	Nd	0.29	Nd
C8:0Me	Nd	Nd	Nd	Nd	Nd
C9:0Me	Nd	Nd	Nd	Nd	Nd
C10:0Me	Nd	Nd	Nd	Nd	Nd
C11:0Me	Nd	Nd	Nd	Nd	Nd
C12:0Me	Nd	Nd	Nd	Nd	Nd
C13:0Me	Nd	Nd	Nd	Nd	Nd
C14:0Me	Nd	Nd	Nd	Nd	Nd
C15:0Me	Nd	Nd	Nd	Nd	Nd
C16:0Me	Nd	Nd	Nd	Nd	Nd
C17:0Me	Nd	Nd	Nd	Nd	Nd
C18:0Me	Nd	Nd	Nd	Nd	Nd
C20:0Me	Nd	Nd	Nd	Nd	Nd
C21:0Me	Nd	Nd	1.6	1.0	2.0
C22:0Me	0.08	Nd	3.1	Nd	Nd
C16:1cMe	Nd	0.01	Nd	Nd	Nd
C18:1tMe	Nd	Nd	Nd	Nd	Nd
C18:1cMe	Nd	Nd	Nd	Nd	Nd
C18:2cMe	Nd	Nd	Nd	Nd	Nd
C18:3c6Me	0.85	1.15	7.2	14	8.7
C18:3c9Me	Nd	Nd	Nd	Nd	Nd
C20:5cMe	0.16	Nd	Nd	Nd	Nd

Sum c [µg L^{-1}]	1.1	1.2	13	21	14

References

1. Hartig C. Rapid identification of fatty acid methyl esters using a multidimensional gas chromatography-mass spectrometry database. J Chromatogr A. 2008;1177(1):159-69.
